# Supplementary material for: Time-series transcriptome analysis identified differentially expressed genes in broiler chicken infected with mixed Eimeria species
Source: Front Genet. 2022 Aug 8;13:886781. doi: 10.3389/fgene.2022.886781 (PMC9393255; doi:10.3389/fgene.2022.886781)
Supplement: Supplementary file 2 [file DataSheet1.ZIP › 7dpi_KEGG.Gsea.1625115961950/KEGG_ALANINE_ASPARTATE_AND_GLUTAMATE_METABOLISM.html]

Details for gene set KEGG\_ALANINE\_ASPARTATE\_AND\_GLUTAMATE\_METABOLISM[GSEA]

|  || Dataset | TMM\_7dpi\_gct\_format\_7dpi\_gct\_format.Class\_4dpi.cls #PC\_versus\_NC.Class\_4dpi.cls #PC\_versus\_NC\_repos |
| Phenotype | Class\_4dpi.cls#PC\_versus\_NC\_repos |
| Upregulated in class | 1 |
| GeneSet | KEGG\_ALANINE\_ASPARTATE\_AND\_GLUTAMATE\_METABOLISM |
| Enrichment Score (ES) | 0.35734305 |
| Normalized Enrichment Score (NES) | 1.2832736 |
| Nominal p-value | 0.11820084 |
| FDR q-value | 0.35289583 |
| FWER p-Value | 0.969 |
Table: GSEA Results Summary

  

Fig 1: Enrichment plot: KEGG\_ALANINE\_ASPARTATE\_AND\_GLUTAMATE\_METABOLISM      
 Profile of the Running ES Score & Positions of GeneSet Members on the Rank Ordered List

  

| SYMBOL | TITLE | RANK IN GENE LIST | RANK METRIC SCORE | RUNNING ES | CORE ENRICHMENT || 1 | GOT2 | na | 227 | 1.009 | 0.1143 | Yes |
| 2 | GFPT2 | na | 567 | 0.700 | 0.1785 | Yes |
| 3 | GOT1 | na | 673 | 0.649 | 0.2553 | Yes |
| 4 | GLS2 | na | 687 | 0.641 | 0.3389 | Yes |
| 5 | ASS1 | na | 1192 | 0.458 | 0.3573 | Yes |
| 6 | ALDH4A1 | na | 2002 | 0.271 | 0.3257 | No |
| 7 | DDO | na | 2086 | 0.256 | 0.3526 | No |
| 8 | PPAT | na | 2966 | 0.131 | 0.2966 | No |
| 9 | AGXT2 | na | 3815 | 0.026 | 0.2293 | No |
| 10 | GLUL | na | 4227 | -0.021 | 0.1978 | No |
| 11 | ADSL | na | 4430 | -0.043 | 0.1866 | No |
| 12 | GLUD2 | na | 4781 | -0.078 | 0.1678 | No |
| 13 | IL4I1 | na | 4842 | -0.086 | 0.1741 | No |
| 14 | CPS1 | na | 5072 | -0.110 | 0.1695 | No |
| 15 | CAD | na | 5401 | -0.139 | 0.1605 | No |
| 16 | ALDH5A1 | na | 5533 | -0.152 | 0.1696 | No |
| 17 | ASNS | na | 5766 | -0.177 | 0.1736 | No |
| 18 | ASPA | na | 6350 | -0.239 | 0.1565 | No |
| 19 | GFPT1 | na | 8396 | -0.471 | 0.0482 | No |
| 20 | NIT2 | na | 8538 | -0.490 | 0.1011 | No |
| 21 | GPT2 | na | 9710 | -0.662 | 0.0908 | No |
| 22 | ABAT | na | 10275 | -0.765 | 0.1447 | No |
Table: GSEA details [plain text format]

  

Fig 2: KEGG\_ALANINE\_ASPARTATE\_AND\_GLUTAMATE\_METABOLISM      
 Blue-Pink O' Gram in the Space of the Analyzed GeneSet

  

Fig 3: KEGG\_ALANINE\_ASPARTATE\_AND\_GLUTAMATE\_METABOLISM: Random ES distribution      
 Gene set null distribution of ES for **KEGG\_ALANINE\_ASPARTATE\_AND\_GLUTAMATE\_METABOLISM**
